# Predicting Relapse in Patients With Triple Negative Breast Cancer (TNBC) Using a Deep-Learning Approach

**DOI:** 10.3389/fphys.2020.511071

**Published:** 2020-09-23

**Authors:** Guangyuan Yu, Xuefei Li, Ting-Fang He, Tina Gruosso, Dongmei Zuo, Margarita Souleimanova, Valentina Muñoz Ramos, Atilla Omeroglu, Sarkis Meterissian, Marie-Christine Guiot, Li Yang, Yuan Yuan, Morag Park, Peter P. Lee, Herbert Levine

**Affiliations:** ^1^Department of Physics and Astronomy, Rice University, Houston, TX, United States; ^2^Center for Theoretical Biological Physics, Rice University, Houston, TX, United States; ^3^Department of Immuno-Oncology, City of Hope Comprehensive Cancer Center, Duarte, CA, United States; ^4^Goodman Cancer Research Centre, McGill University, Montreal, QC, Canada; ^5^Department of Oncology, McGill University, Montreal, QC, Canada; ^6^Department of Pathology, McGill University Health Centre, Montreal, QC, Canada; ^7^Department of Surgery, McGill University Health Centre, Montreal, QC, Canada; ^8^Montreal Neurological Institute and Hospital, McGill University, Montreal, QC, Canada; ^9^Department of Medical Oncology and Therapeutics Research, City of Hope Comprehensive Cancer Center, Duarte, CA, United States; ^10^Department of Biochemistry, McGill University, Montreal, QC, Canada; ^11^Department of Bioengineering, Northeastern University, Boston, MA, United States; ^12^Department of Physics, Northeastern University, Boston, MA, United States

**Keywords:** triple negative breast cancer (TNBC), relapse prediction, immunofluorescence images, tumor-infiltrating T cells, machine-learning

## Abstract

The abundance and/or location of tumor infiltrating lymphocytes (TILs), especially CD8^+^ T cells, in solid tumors can serve as a prognostic indicator in various types of cancer. However, it is often difficult to select an appropriate threshold value in order to stratify patients into well-defined risk groups. It is also important to select appropriate tumor regions to quantify the abundance of TILs. On the other hand, machine-learning approaches can stratify patients in an unbiased and automatic fashion. Based on immunofluorescence (IF) images of CD8^+^ T lymphocytes and cancer cells, we develop a machine-learning approach which can predict the risk of relapse for patients with Triple Negative Breast Cancer (TNBC). Tumor-section images from 9 patients with poor outcome and 15 patients with good outcome were used as a training set. Tumor-section images of 29 patients in an independent cohort were used to test the predictive power of our algorithm. In the test cohort, 6 (out of 29) patients who belong to the poor-outcome group were all correctly identified by our algorithm; for the 23 (out of 29) patients who belong to the good-outcome group, 17 were correctly predicted with some evidence that improvement is possible if other measures, such as the grade of tumors, are factored in. Our approach does not involve arbitrarily defined metrics and can be applied to other types of cancer in which the abundance/location of CD8^+^ T lymphocytes/other types of cells is an indicator of prognosis.

## Introduction

In nearly all cancer types, it has been demonstrated that patients with higher numbers of tumor infiltrating lymphocytes (TILs) in their solid tumors usually have better prognosis in term of the overall survival as well as the disease-free survival (Gooden et al., 2011). Most studies focused on CD8^+^ T lymphocytes (Sato et al., 2005; Galon et al., 2006; Sharma et al., 2007; Mahmoud et al., 2011; Rahbar et al., 2015; Carstens et al., 2017), which can recognize and kill cancer cells with specific antigens (Martínez-Lostao et al., 2015). For example, in colorectal cancer and melanoma (Pagès et al., 2009; Galon et al., 2016), the ratio of T-cell density in the core of a tumor (CT) to that at the invasive margin (IM), i.e., the Immunoscore, has demonstrated its power to indicate prognosis.

However, due to the heterogeneity of the abundance of TILs within tumors, selection of the threshold-value for defining patient categories can be ambiguous. Furthermore, the exact threshold-value as well as the choice of a suitable metric (such as the Immunoscore defined in colorectal cancer) can vary from one type of cancer to another. In order to reduce such ambiguities, a machine-learning approach can be helpful due to its parameter-free formulation. Indeed, there have recently been a few successful applications of machine-learning approaches in cancer research: Agarap (2017) compared six machine-learning (ML) algorithms on the Wisconsin Diagnostic Dataset for a binary prediction problem of benign vs. malignant tumor; Heidari et al. (2018) developed a machine-learning approach to predict short-term cancer risk by comparing asymmetry of the left vs. right breasts; Saltz et al. (2018) trained a convolutional neural network (CNN) to recognize TILs in the H&E histological images from the TCGA database and generated TIL maps of TCGA samples; here the authors showed that TIL densities and spatial structure can be associated with features such as tumor types, immune subtypes, and tumor molecular subtypes.

In this work, using immunofluorescence (IF) images of CD8^+^ T lymphocytes and cancer cells, we developed a machine-learning approach to predict the risk of relapse for patients with Triple Negative Breast Cancer (TNBC). We first used tumor-section images of 24 patients with either poor or good outcome to train a specific convolutional neural network (CNN) called MXNet. Subsequently, the trained CNN was applied to predict whether a patient is expected to have a good or poor outcome in an independent test set. This test set is a distinct cohort of TNBC patients (29 of them) from a different medical center.

**FIGURE 1:**
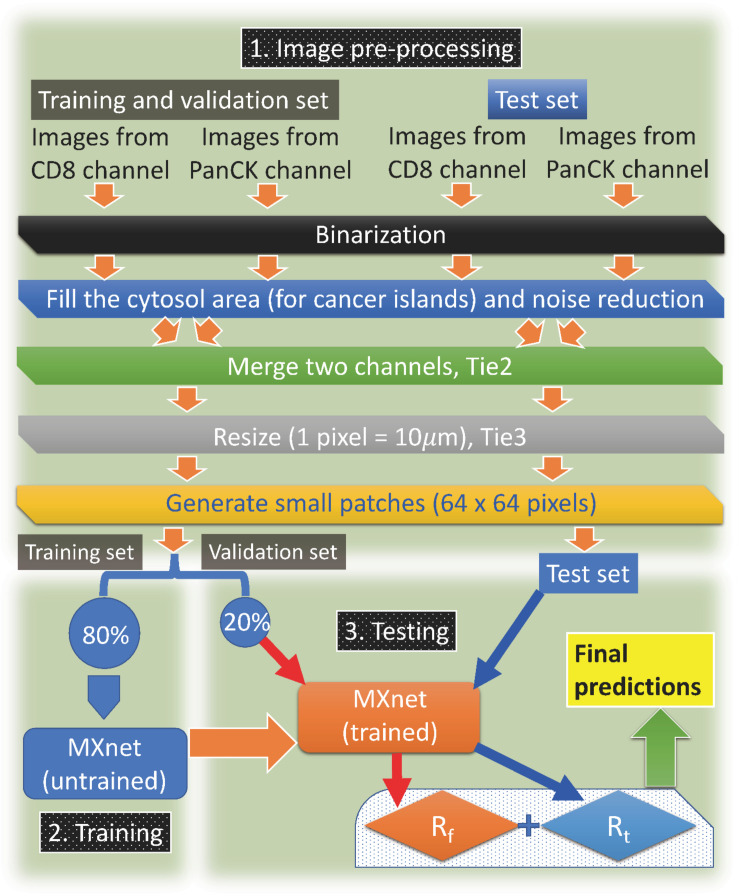
The flowchart. Overall description of our machine-learning approach.

An overall workflow of our approach is shown in Figure 1. Our results, to be detailed below, show that the 6 patients (out of 29) who belong to the poor outcome group are all correctly predicted by our procedure; for the 23 patients (out of 29) who belong to the good-outcome group, 17 of them are correctly predicted. This number might increase if additional factors such as tumor grade or nodal involvement are taken into account.

Compared to other metrics, such as the overall CD8^+^ T-cell density or the infiltration level into tumor islets of CD8^+^ T cells, we show that our machine-learning approach has better predictive power. Due to the automatic nature of our procedure, we believe this approach could be readily applied to other types of cancer where the abundance/location of CD8^+^ T lymphocytes (or any type of non-cancer cells) is likely to be an indicator of prognosis. Furthermore, our algorithm does not rely on clinical training or experience, which means this method could be widely adopted.

## Materials and Methods

### Patients and Specimens

There are two independent cohorts in our study: one from the City of Hope (CH, 24 patients in total) and the other from McGill University (MG, 29 patients in total). All these patients had TNBC and underwent surgery. All 55 patients were treatment-naive before the surgery. Details of the sample collection for the two cohorts are described in the following.

For the City of Hope cohort, samples from patients diagnosed with triple-native breast cancer, invasive ductal carcinoma (IDC) type, and treated at COH from 1994 to 2015 were retrieved. At the time of surgery, none of the patients had prior treatment. Archived formalin-fixed paraffin-embedded (FFPE) tumor tissues were sectioned into 5 μm thick slides and baked onto glass microscope slides and labeled with anti-pan cytokeratin (AE1/AE3, Dako) and anti-CD8 (SP16, Biocare) using the Opal TSA system (Akoya Bioscience). Stained samples were further counterstained with DAPI, cover-slipped with ProLong^®^ Gold Antifade mounting media, and imaged by Vectra automated imaging system.

For the McGill cohort, it is a subset of the cohort published in Gruosso et al. (2019). Samples were collected from patients undergoing breast surgeries at the McGill University Health Centre (MUHC) between 1999 and 2012. All tissues were snap-frozen in O.C.T. Tissue-Teck Compound within 30 min of removal. For the purposes of this study, samples were selected according to the following criteria: therapy-naive at time of surgical excision, clinically documented lack of expression/amplification of ER, PR and HER2, a histological subtype assignment of invasive ductal carcinoma [not otherwise specified) (IDC (NOS)] and availability of matched formalin-fixed paraffin-embedded (FFPE) tumor blocks. Information regarding clinical variables and disease course (follow-up) was obtained through review of Medical Records at the MUHC. Five micro meter sections from frozen tissue were prepared for each sample, subjected to routine hematoxylin and eosin (H&E) staining, and evaluated by an attending clinical pathologist with expertise in breast tissue to identify invasive, *in situ* and normal components. Cancer cells and CD8^+^ T cells were labeled by pan-cytokeratin (PanCK) and CD8 immune-fluorescence (IF) antibodies, respectively. Primary antibodies for immunofluorescence (IF) as well as the IF protocol were described and detail in Li et al. (2019).

Patients were divided into two outcome-groups: any patient who had a relapse within 3 years of the surgery belongs to the poor-outcome group; conversely, any patient who survived and did not have a relapse within 5 years belongs to the good-outcome group. Apparently, there is 1 year gap between the good and poor outcome groups and rare individuals that fall in-between are dropped. We chose this particular standard because we want to ensure that patients in the two groups are well-separated.

### Pre-processing of the Immunofluorescent Images

The original resolution of our images is 0.5 μm (CH) and 0.975 μm (MG), respectively. We first binarize the two IF channels for all images: pixels with an IF intensity in the top 90% (among all pixels within an individual image) are assigned as 1 and others are assigned as 0. Then the binary images of cancer cells are labeled in white (1) and black (0) and the binary images of CD8^+^ T cells are labeled in red (1) and black (0). In addition, we remove the isolated connected-areas with an area smaller than 200 μm^2^, which might be due to the noise in the IF signals. Furthermore, for the binary images of cancer cells, since PanCK is a cell-surface marker, the cytosol of cancer cells might be black. In order to faithfully represent the area of cancer islets, we automatically fill holes with an area smaller than 200 μm^2^. The two corresponding binary images for cancer cells and CD8^+^ T cells are subsequently merged together to generate images (Tie2) for further processing.

For deep-learning, the suitable image-size is usually 32 to a few hundred pixels in one dimension, while our original images can be around 20,000 pixels in on dimension. Therefore, we resize all Tie2 images to the same scale for the two independent cohorts so that 1 pixel in each image (Tie3) corresponds to 10 μm. Next, each image (Tie3) is divided into smaller (adjacent) patches (64 × 64 pixels). If the patch has a number of white (PanCK^+^) pixels that is less than a quarter of the total number of pixels in this patch, it will be discarded. If the patch has no CD8^+^ pixels in it, it will also be discarded. After this step, we now have final images (Tie4) for the machine-learning procedure later. Note that according to our standard, some of the areas at the invasive margin of a tumor might be discarded because of the lack of cancer cells, though some parts of the invasive margins are kept in the analysis. An example illustrating the areas kept in the analysis is shown in Supplementary Figure S1. Furthermore, in the Supplementary Material, we tested the effects of changing the spatial resolution of patches in detail (Supplementary Table S1). The results indicate that our baseline procedure is optimal for the current dataset. Finally, we also demonstrated that discarding patches without T cells does not substantially change our original results (Supplementary Table S2).

### Training

For the training set, we use patches from patients in the CH cohort. A detailed table of the total number of small patches for each patient in the training set can be found in Table 1. Briefly, there are 9 patients with the poor outcome and 15 patients with the good outcome.

**TABLE 1:** Number of patches derived from the images of the training set and clinical information.

ID	Patch #	Outcome	Rtn	Grade	Nodal-status
P1	44	Good	0.17–0.41	III	No
P2	811	Good	0.92–0.97	III	No
P3	810	Good	0.87–0.99	III	No
P4	220	Good	0.98–1	III	No
P5	834	Good	0.99–1	III	NA
P6	226	Good	0.93–1	III	No
P7	171	Good	0.72–0.84	II	Yes
P8	92	Good	0.77–1	III	No
P9	30	Good	0.80–0.87	III	No
P10	387	Good	0.68–0.77	III	No
P11	471	Good	0.75–0.94	III	Yes
P12	228	Good	1	III	No
P13	260	Good	0.29–0.58	II	No
P14	30	Good	0.33–0.59	II	NA
P15	243	Good	0.31–0.45	III	Yes
P16	218	Poor	0	III	No
P17	84	Poor	0–0.22	III	No
P18	129	Poor	0.04–0.31	NA	Yes
P19	82	Poor	0.06–0.11	III	No
P20	290	Poor	0–0.02	III	No
P21	113	Poor	0–0.06	III	No
P22	144	Poor	0.08–0.24	III	Yes
P23	256	Poor	0.13–0.22	III	No
P24	235	Poor	0–0.08	III	No

**Column 4 is range of R_tn_ derived from the validation set (20% of patches from each patient in the CH cohort) after 5 rounds of testing using randomly selected patches. For each patient, R_tn_ is the percentage of the patches that is predicted to be from patients with the good outcome.**

**FIGURE 2:**
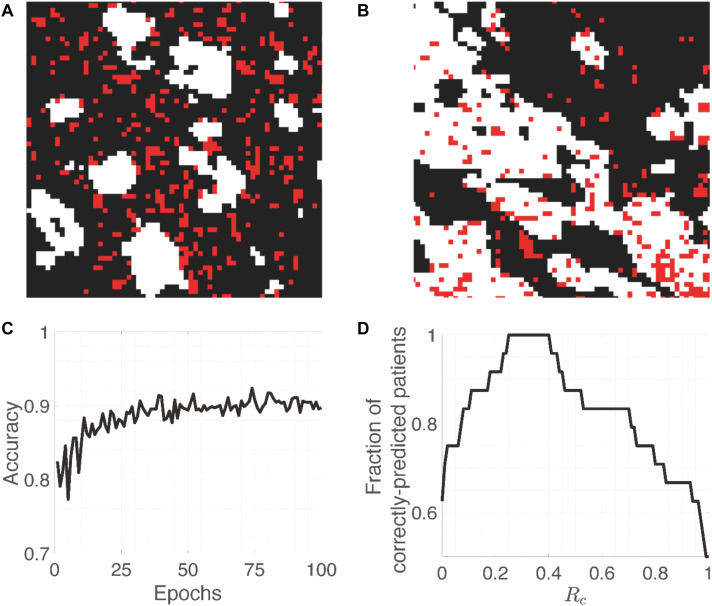
Training images and accuracy. **(A,B)** Representative patches (64 × 64 pixels) from patients with the poor and good outcome, respectively. While and red pixels represent PanCK-positive (cancer cells) and CD8-positive (CD8^+^ T cells) areas, respectively. **(C)** Evolution of the accuracy on training patches as a function of Epochs. **(D)** The fraction of correctly-predicted patients as a function of the cut-off percentage (R_c_) of patches that are classified as arising from a patient with the good outcome.

For the CH cohort, 80% of the small patches from each patient are used for training. The other 20% are used for the threshold-selection procedure (validation) described later. There are more patches from patients with the good outcome in the training set (4857 vs. 1551); hence, in order to make the training balanced between samples from poor and good outcomes, we generated 3 additional copies of each small patch from patients with the poor outcome and added them to the training set. Examples of patches are shown in Figures 2A,B. In addition, to test whether generating additional copies would bias the model prediction, we investigated other methods of balancing the number patches from the two prognostic groups in the training set (Supplementary Table S3). The details can be found in the (Supplementary Table S4), and there was no substantial difference between the two balancing methods, thus overfitting was less of a concern.

For the deep-learning network, we use “deepflow” from MXNet (Chen et al., 2015; Krizhevsky et al., 2017) for this project. The code that we developed can be found at https://github.com/xun6000/deepflow. Note that the procedure to feed these images into MXNet is a bit complex, and the github file contains the command line instruction to do so properly. In the following, we will describe the procedure of the training algorithm.

With one input small patch (64 × 64 pixels), a probability can be computed by MXNet to determine whether this patch is from a patient with the poor outcome. Since we know *a priori* where this patch comes from, based on the difference between this probability and its known value (0 for the good outcome or 1 for the poor outcome), the internal parameters of MXNet are updated automatically using the optimization algorithm called RMSProp (Ruder, 2016). We choose the input parameters for RMSProp as learning_rate = 0.0005, weight_decay = 0.01, factor_epoch = 10, lr_factor = 0.25. In addition, the mini-batch size for RMSProp is related to the performance of network, i.e., larger mini-batch size will make the net harder to find the global minimum (Keskar et al., 2016). The mini-batch size is the number of images that are fed together to MXNet for one round of update for the internal parameters in MXNet. Specifically, we use 20 images as our mini-batch size.

One epoch is defined as the process in which all patches were served as the input to train the MXNet based on the defined outcome (the other input information). We run 100 epochs to train the MXNet after which the accuracy should have been stabilize (Figure 2C). If we select the cut-off probability between a poor-outcome patch and a good-outcome one to be 0.5, i.e., probability >0.5 means that the patch is from a patient with the poor outcome, then the training and validation accuracy are around 0.9 and 0.85, respectively. This is understandable because individual patches from patients with the good outcome may resemble those from patients with the poor outcome, and vice versa. The whole training process takes 3–4 h on a Tesla K80 NVIDIA GPU.

## Results

### Patient Stratification Criteria Based on Deep-Learning Predictions

After training, the network can predict whether a small patch is from a patient with the good or poor outcome, which is named as a “good” or “poor” patch. We then used the trained CNN to predict the remaining small patches (the 20% mentioned before) so that we can determine the percentage (Rtn) of the “good” patches in each patient. For a given cut-off percentage Rc, we discover whether Rtn of a patient is higher or lower than Rc. If we assume that any patient whose Rtn < Rc is predicted to have the poor outcome (and vice versa), we will achieve some degree of accuracy of the prediction (Ac) by the trained MXNet. We then change Rc until Ac reaches the maximum, selecting the percentage (Ropt) that best-separates the two groups of the 24 patients in the training cohort.

Since the 20% of small patches from the CH cohort are randomly selected and the CNN can also have some randomness, the Rtn for each patient can vary for different realizations (column 4, Table 1). Furthermore, for each realization, there is a range of Rc that gives the same accuracy. After going through 5 realizations, we find the Ropt should be between 0.14 and 0.40. Most of the times (4 out 5), we can find a Ropt that makes a perfect separation (Figure 2D). Specifically, we select the average of the values that can give a perfect separation in those 5 realizations, which is Rc =0.30.

### Predicted Prognosis for the Independent Cohort

Next, the percentage (Rt) of good patches can be determined for each patient in the test set (MG cohort). Thus, these patients will be predicted to have the poor (Rt < Rc) or good (Rt > Rc) outcome. Our results show that for the 6 patients (out of 29) who belong to the poor-outcome group, they are all correctly predicted by our approach; for the 23 patients (out of 29) who belong to the good-outcome group, 17 of them are correctly predicted (Table 2).

**TABLE 2:**
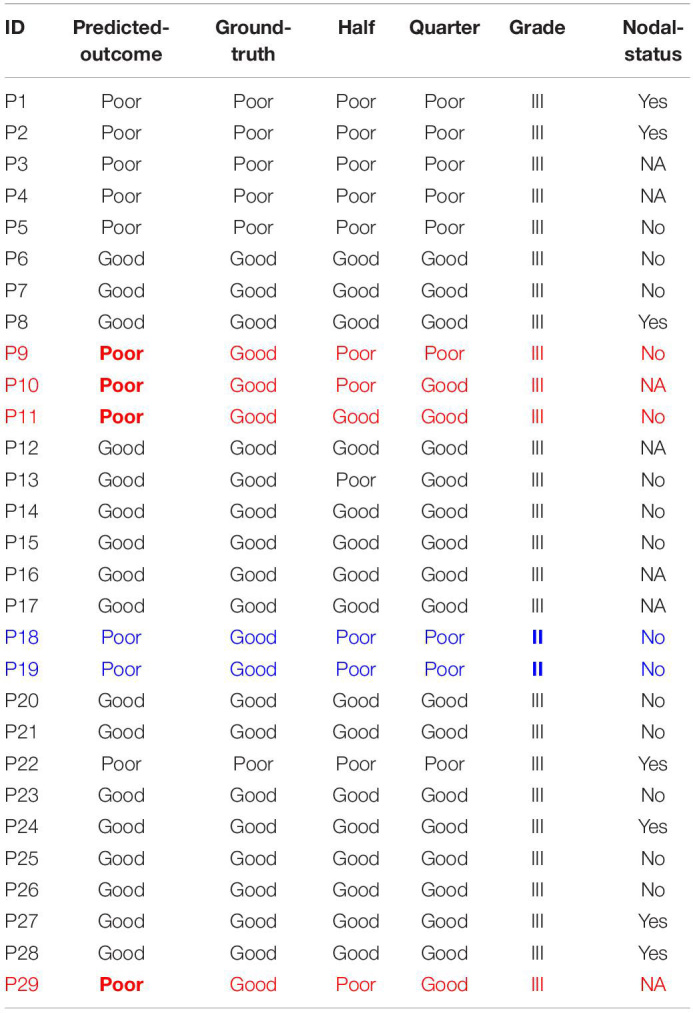
Predicted vs. actual outcome for the test set (MG cohort).

**The actual outcome of individual patient is shown in column 3. Prediction of our machine-learning approach using the full-, half-, and quarter-size section samples are shown in columns 2, 4, and 5. Information on the Grade and Nodal status of the tumors is shown in columns 6 and 7. Rows that are labeled in blue are for patients with Grade II tumors. Rows that are labeled in red are for patients with a poor-outcome prediction (column 2) but a good-outcome in reality (column 3). NA stands for not applicable.**

Furthermore, the imperfect prediction could be due to factors other than the CD8^+^ T cells. For example, if we also integrate other clinical information such as the nodal status and the tumor grade, for the 6 patients that are not correctly predicted by our approach, there are 2 of them (Patients 18 and 19) whose tumor grade is lower (grade II). Note that all other 27 patients in the MG cohort have grade III tumors (Table 2); and all 3 patients with grade II tumors in the CH cohort belong the good-outcome group (Table 1). In addition, it is valuable to notice that the Nodal status of the other 4 incorrect predictions (rows highlighted in red in Table 2) is either No or NA, whereas the Nodal status of correctly-predicted poor-outcome patients is mostly Yes or NA with only one exception (P5) out of 6 patients (P1–P5 and P22). However, in the poor-outcome group of the training cohort (CH), only 2 out of 9 patients have a positive Nodal status. Therefore, combining the prediction using our approach with the information on the tumor grade and the nodal status, the accuracy might be improved significantly. This needs to be tested in the future for a data-set with more complete annotation regarding Nodal status.

In addition, we tested the degree to which the accuracy of our prediction is diminished if the size of the section samples decreases to half or a quarter of the original samples (columns 4 and 5 in Table 2). For 3 of the patients (out of 29), because of the inhomogeneity of the tumors, the prediction is not perfectly robust to the region of selection.

When defining poor outcome as positive, the confusion matrix equals as follows:

**Table:** 

	ground truth 1	ground truth 0
predict 1	6	6 (Type II error)
predict 0	0 (Type I error)	17

The recall = 6/6 = 1, precision = 6/12 = 0.5

### Comparison of the Prediction-Accuracy Between the Deep-Learning Method and CD8^+^ T-Cell Number or Infiltration Level

**FIGURE 3:**
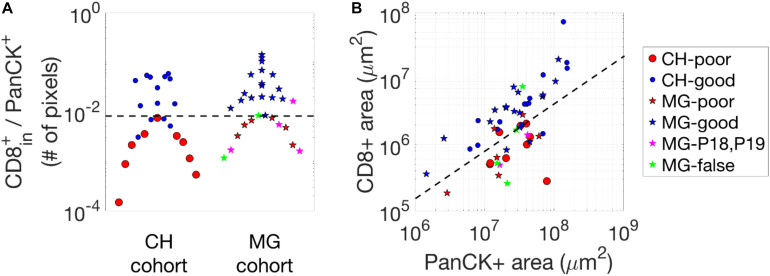
Statistics of CD8^+^ pixels in images. **(A)** The density of CD8^+^ pixels inside cancer-cell islands, i.e., the number of CD8 + pixels divided by the number of PanCK^+^ pixels. **(B)** CD8-positive and PanCK-positive areas (in μm^2^) of the two cohorts. The black dash lines in each figure are selected manually to separate the groups (poor- vs. good-outcome) of the patients which give rise to the highest accuracy when comparing to the actual outcome.

The machine-learning approach gives a reasonably good prediction of the 3 year relapse likelihood. We tried to compare the accuracy of this prediction with other possible metrics, such as the density of CD8^+^ T cells inside cancer-cell islands, the absolute numbers of CD8^+^ T cells and cancer cells, etc. In Figure 3, there exists an apparent overlap between the poor- and good-outcome group using the density of CD8^+^ T cells inside cancer-cell islands (Figure 3A) or the absolute numbers of CD8^+^ T cells and cancer cells (Figure 3B). Note that for the CH cohort, using our deep-learning approach (Figure 2D), we can have a perfect separation between the two groups of patients. Nevertheless, if we manually select the “perfect” cut-offs according to the data, as demonstrated by the dash lines in Figure 3, the maximum stratification accuracy considering the density of CD8^+^ T cells inside cancer-cell islands or the absolute numbers of CD8^+^ T cells and cancer cells will be 85 and 87%, respectively. Even though the accuracy using these methods is comparable to our deep-learning approach, the selection of the cut-off is not statistically justified.

To further test whether other clinical data could better predict the outcome, we performed hierarchical clustering and principal component analysis based on the clinical characteristics collected for the CH cohort (see Supplementary Tables S5, S6). In short, these analyses did not give an adequate separation of the two prognostic groups, whereas our current baseline procedure was successful. More details are provided in section 5 of the Supplementary Material.

### Information Extracted by Our Machine-Learning Approach in Determining the Outcome

**FIGURE 4:**
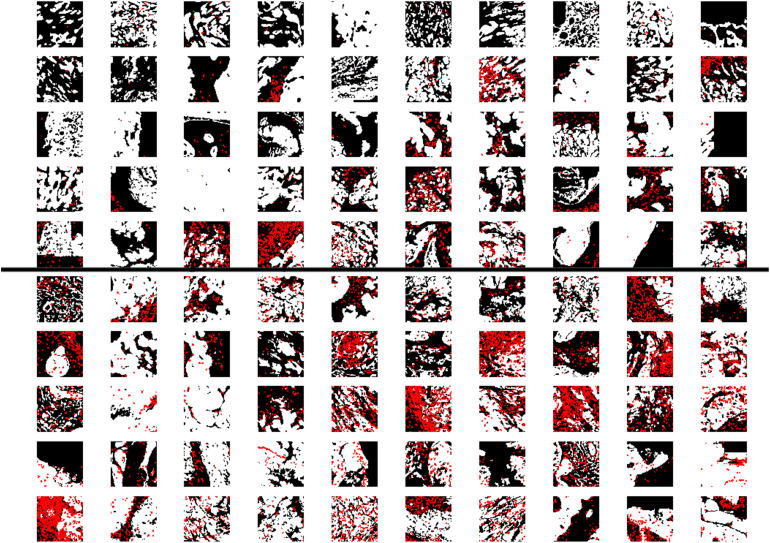
Examples of patches. Patches that are predicted to be from patients with the poor outcome (upper 50) or good outcome (lower 50). The size of each patch is 64 pixels (64 pixels, with 1 pixel = 10 μm White and red pixels represent PanCK-positive (cancer cells) and CD8-positive (CD8^+^T cells) areas, respectively. For the patches in the upper 50, we still observe many samples have a lot of red pixels (CD8-positive areas), however, compared to patches in the lower 50, most of the red dots are in the tumor stroma (black areas) instead of cancer-cell islands (white areas).

Finally, we describe the information extracted by our machine-learning approach in determining the outcome. The results suggest that the absolute density or number of CD8^+^ T cells might not be the most important factor but instead the (relative) infiltration of CD8^+^ T cells is more crucial. As demonstrated in Figure 4, we generally observe that patches from patients with the good outcome have more red pixels (CD8^+^) as compared to their counterparts. However, for patches from patients with poor outcome, we can still observe patch samples with many CD8^+^ T cells (red pixels) but these pixels are outside of the cancer islands (white areas); and we observe patches with fewer red pixels (CD8^+^ T cell) but most of them are inside white areas (cancer islands), from patients with the good outcome. In summary, our results indicate that the relative infiltration level of CD8^+^ T cells into cancer-cell islands is the most important factor to determine whether a patch would be predicted to arise from a patient with the good outcome.

## Discussion

In this work, we developed a machine-learning approach to predict the 3 year relapse likelihood based on IF images of cancer cells and CD8^+^ T cells. While the approach is effective with an accuracy 86% or higher, there is still room to further improve the accuracy of our approach by including additional features that can be measured in addition to CD8 makers. In the following, we will further discuss possible candidates.

First, the molecular states of CD8^+^ T cells can be diverse (Guo et al., 2018), including different levels of exhaustion (Wherry and Kurachi, 2015). Therefore, it would be more informative to also assess the functional states of individual CD8^+^ T cells via additional markers, such as Granzyme B, EOMES, T-bet, PD-1, and so on. By incorporating these additional features of CD8^+^ T cells, the prediction-accuracy of our approach could be further improved.

Secondly, there are other types of immune cells beyond CD8^+^ T cells that have been demonstrated to have predictive power in patient prognosis, such as tumor-associated macrophages (Zhang et al., 2012) as well as Tregs (Shang et al., 2015). Having an image with the information regarding several types of these immune cells again might improve the accuracy of our approach.

Thirdly, properties of cancer cells also matter in predicting outcome, in addition to the spatial information of cancer cells and CD8^+^ T cells. For example, in our test cohort, we found that two good-outcome patients who are predicted to have poor outcome actually have Grade II tumors, where the proliferation rate of tumor cells is low. In fact, all patients with Grade II tumors belong to the good-outcome group. Another related possibility still to be investigated is the EMT status of cancer cells. This is motivated by the fact that markers for epithelial-to-mesenchymal transition (EMT) of cancer cells are usually indicative for progression of disease (Tsoukalas et al., 2017; Luo et al., 2018).

The current formulation of our algorithm is a binary class problem in nature. We might imagine changing this binary class problem to a triple class problem, where the 3rd class is the patches that we currently discarded. This can make the application of the algorithm much simpler. The details can be found in the Supplementary Material.

Our final remark concerns one weak point of our approach. Due to the heterogeneity of tumors, it is not currently possible to accurately predict the outcome of a patient based on a small sub-sample of one part of a tumor. For example, for P13 shown in Table 2, using half of the patches from the tumor gives the opposite prediction, compared to using a quarter or the whole section. Changing the location of the selection of the half can also change the prediction; again this is due to the heterogeneity of the tumor itself. Therefore, to predict prognosis based on a limited number of patches, it is important to sample multiple sites of a tumor instead of from only one part.

In summary, we developed a machine-learning approach that can predict the 3 year relapse risk of TNBC based on the IF images of cancer cells and CD8^+^ T cells, with an accuracy 86% or higher. The advantage of this approach is that the standards to determine outcome are relatively objective. Therefore, it can readily be applied to other types of samples. With more training samples and more features measured, this approach should reach even higher prediction accuracy and become useful for rapid clinical prognosis.

## Data Availability Statement

The original images used and analyzed in this study can be accessed via the link provided in the ‘data.txt’ file on Github (https://github.com/xun6000/deepflow/blob/master/data.txt).

## Ethics Statement

The studies involving human participants were reviewed and approved by the City of Hope Institutional Review Board (IRB #14346) via the City of Hope Biospecimen Repository McGill University Health Centre (MUHC) review board (study approval SUR-2000-966 and SUR-99-780). The patients/participants provided their written informed consent to participate in this study.

## Author Contributions

GY, XL, and HL designed the study. AO, SM, M-CG, YY, and PL included and followed patients. T-FH, MS, and VR provided clinical data. T-FH, DZ, and TG performed the IHC analyses. GY and XL developed the algorithm. LY developed the triple-class algorithm. MP, PL, and HL supervised the study. GY, XL, T-FH, TG, MP, PL, and HL wrote the manuscript. All coauthors read and approved the final manuscript.

## Conflict of Interest

The authors declare that the research was conducted in the absence of any commercial or financial relationships that could be construed as a potential conflict of interest.
