# Supplementary material for: Predicting Relapse in Patients With Triple Negative Breast Cancer (TNBC) Using a Deep-Learning Approach
Source: Front Physiol. 2020 Sep 23;11:511071. doi: 10.3389/fphys.2020.511071 (PMC7538858; doi:10.3389/fphys.2020.511071)
Supplement: Supplementary file 1 [file Table_1.DOCX]

**Supplemental Material: Predicting relapse in patients with Triple Negative Breast Cancer (TNBC) using a deep-learning approach**

Guangyuan Yu^1,2,*^, Xuefei Li^2,13,*^, Ting-Fang He^3^, Tina Gruosso^4,5^, Dongmei Zuo^4^, Margarita Souleimanova^4^, Valentina Muñoz Ramos^4^, Atilla Omeroglu^6^, Sarkis Meterissian^5,7^, Marie-Christine Guiot^6,8^, Li Yang^1^,Yuan Yuan^9^, Morag Park^4,5,10,+^, Peter P. Lee^3,+^, Herbert Levine^11,12,+^

1. Department of Physics and Astronomy, Rice University, Houston, TX 77030
2. Center for Theoretical Biological Physics, Rice University, Houston, TX 77030
3. Department of Immuno-Oncology, City of Hope Comprehensive Cancer Center, Duarte, CA 91010
4. Goodman Cancer Research Centre, McGill University, Montreal, QC H3A 1A3, Canada
5. Department of Oncology, McGill University, Montreal, QC H4A 3T2, Canada
6. Department of Pathology, McGill University Health Centre, Montreal, QC H4A 3J1, Canada
7. Department of Surgery, McGill University Health Centre, Montreal, QC H4A 3J1, Canada
8. Montreal Neurological Institute and Hospital, McGill University, Montreal, QC H3A 2B4, Canada
9. Department of Medical Oncology & Therapeutics Research, City of Hope Comprehensive Cancer Center, Duarte, CA 91010
10. Department of Biochemistry, McGill University, Montreal, QC H3A 1A3, Canada
11. Department of Bioengineering, Northeastern University, Boston, MA 02115
12. Department of Physics, Northeastern University, Boston, MA 02115
13. Present address: Shenzhen Institute of Synthetic Biology, Shenzhen Institutes of Advanced Technology, Chinese Academy of Sciences, Shenzhen, Guangdong 518055, China

^*^ These authors contributed equally to this work

^+^ To whom correspondence should be addressed: Morag Park <morag.park@mcgill.ca>, Peter Lee <plee@coh.org>, and Herbert Levine <h.levine@northeastern.edu>

Tel: +1 (617) 373 2902
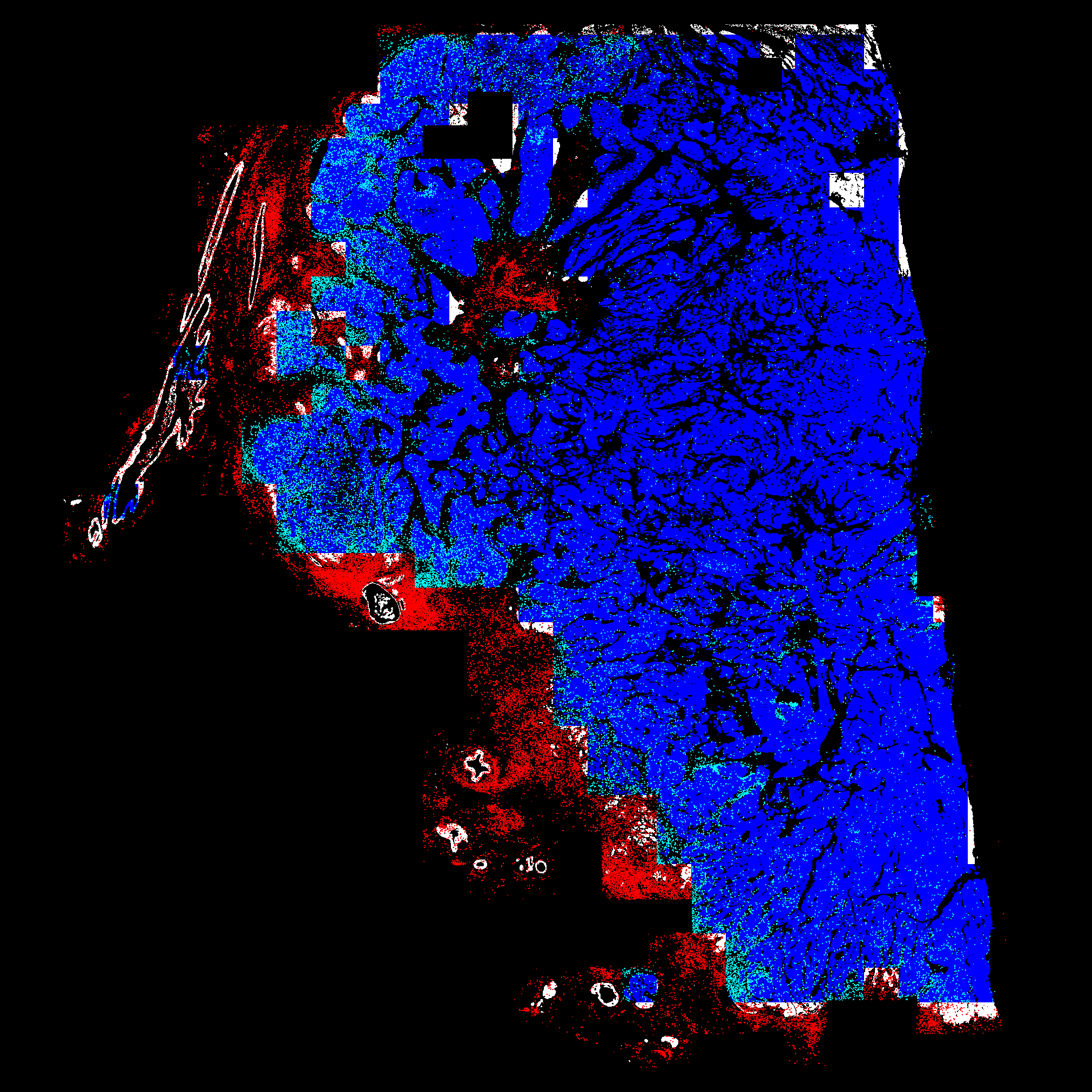


**Figure S1:** An example (patient 10 in the CH cohort) demonstrates the areas that are used in the machine-learning analysis. As described in the main text, the whole section image is divided into smaller (adjacent) patches (64 pixels x 64 pixels). If the patch has a number of PanCK^+^ pixels that is less than a quarter of the total number of pixels in this patch, it will be discarded. If the patch has no CD8^+^ T cell pixels in it, it will also be discarded. According to this standard, the tumor areas that are kept (or discarded) in the analysis are labelled in blue (or white) in this figure. And the CD8^+^ T cell pixels that are kept (or discarded) in the analysis are labelled in cyan (or red) in this figure. Note that some of the areas at the invasive margin of a tumor are kept in the analysis.

**1. Effects of varying the size of the patch.**

In our 64*64 pixels patch, 1 pixel equals to 10 µm. To test the effects of changing the scale of the patch, we first cut the whole tumor image into patches with 32 * 32 pixels, and then resize each of them into 64*64 pixels. Now the new patch will still be 64x64, however each pixel corresponds to 5 µm. Secondly, we cut the whole tumor image into patches with 128*128 pixels, and then resize each of them into 64*64 pixels, with the size of the pixel corresponds to 20 µm. Following this procedure, we do not need to change the structure of the original neural network and the remaining steps are the same as the original ones demonstrated in the main text.

For the zoom-in operation (1 pixel = 5 µm), we have 21k patches in the training and validation set and 22 k images in the test set. Carefully tuning the threshold Rc still cannot lead to better predictive power than the original results, and now the predicted outcome are wrong for 9 patients (6 for the original results, out of 29). If we do not tune the threshold, 13/29 will be wrong. Details are presented in Table S1.

For the zoom-out operation (1 pixel = 20 µm), we have 1.6k patches in the training set and 1.9k in the testing set. If we follow the original procedure explained in the main text, 20% of patches as the validation set, that is to say, only about 300 patches will be used to select the threshold Rc and we also find that some patients only have 3 patches in the validation set. The selection of Rc would highly depend on patches selected as the validation. Therefore, the selection of the threshold in such set up will not be reasonable. Nevertheless, we tried to follow the procedure and tested the predictive power with different threshold (0.25,0.3,0.35). Though the number of wrongly predicted patients is comparable to the original results shown in the main texts, we should point out that now some patients with the poor outcome will be predicted to have the good outcome. Again, the detailed findings can be found in Table S1. In term of usefulness, we think the zoom-out version is not better than the original set up, since we might want a stringent screening instead of missing the true positive ones. In addition, some wrongly predicted patients (ground-truth: good outcome) by the original approach now get correct prediction. But confusingly, some of these patients have low infiltration of CD8^+^ T cells, such as P19 (Table S1), therefore, it is difficult to summarize any useful information from the images based on the predicted outcome and considering the limited numbers in the training set, we prefer to maintain the current scale of the patch, which is around 10 µm per pixel.

Comparing these two experiments, we would argue the patch size has two kinds of impact. The first impact is information. There can be many kinds of black areas, some lie outside the tumor, some lie in the stroma between the tumor islands, and some black areas can be found inside the tumor island. 320 µm x 320 µm (5 µm x 64 pixels) is a smaller range. It is harder to tell which kind of environment the CD8^+^ T cell is in. If you see a CD8^+^ T cell in a black area, with a larger image size you are more likely to know which kind of black area it is in, and the classifier will be easier to train. From this perspective, 5-µm resolution is really too small, 10 to 20 µm would be a good range. The second impact is in the number of images. The resolution at 20 µm per pixel will surely lead to fewer patches. In this case, you would not have enough images for training and validation set to give you a threshold. And, for a patient who only has 10 patches in the test set, your prediction would be less confident compared with having 40 patches. It is for these reasons that using patches with the resolution at 5 µm per pixel has very poor predictive power, and using patches with the resolution at 20 µm per pixel can sometimes mistakenly predict a poor patient to be good, which is clearly undesirable. Thus for the data in hand, 10 µm per pixel is the rational choice.

**2. Model predictions based on keeping patches without T cells.**

First, we compared the number of patches with or without discarding patches without T cells: the training set + validation set has 6% more images (CH cohort) and the test set (MG cohort) has 19% more images. Therefore, we in fact retained a large majority of the tumor section image in the original procedure.

Nevertheless, we re-trained the network with the updated set of patches and re-did the predictions. Basically, keeping the patches without T cells brings down the threshold Rc in the validation set. If we use the original threshold 0.35, we will get 9 wrong predictions in the test set (MG cohort), whereas using 0.25, we will get 6 wrong predictions (P9, P10, P11, P18, P19, P29), which is exactly the same as we found in the case of discarding patches without T cells. We think this result is simply explainable. Basically, the patches without T cells should be mainly from patients with the “poor” outcome. For a trained classifier, if we have these “discarded” patches, we will have more poor-outcome patches. The ratio between good/(good+poor) will be smaller. Therefore, for patients with good outcome, the lower bound of good/(good+poor) will be smaller, and for patients with the poor outcome, its upper bound will also be smaller, which then would make the optimal threshold Rc smaller. Therefore, discarding patches without T cells does not substantially change our original results. Details are presented in Table S2.

| CH cohort | | MG cohort | | | | |
| --- | --- | --- | --- | --- | --- | --- |
| validation_32_0.25 | validation_32_0.35 | predict_32_0.25 | predict_32_0.35 | predict_128_0.25 | predict_128_0.3 | predict_128_0.35 |
| (1, 'good') | **(1, 'poor')** | (1, 'poor') | (1, 'poor') | **(1, 'good')** | **(1, 'good')** | **(1, 'good')** |
| (2, 'good') | (2, 'good') | (2, 'poor') | (2, 'poor') | **(2, 'good')** | **(2, 'good')** | (2, 'poor') |
| (3, 'good') | (3, 'good') | (3, 'poor') | (3, 'poor') | **(3, 'good')** | (3, 'poor') | (3, 'poor') |
| (4, 'good') | (4, 'good') | (4, 'poor') | (4, 'poor') | **(4, 'good')** | **(4, 'good')** | **(4, 'good')** |
| (5, 'good') | (5, 'good') | (5, 'poor') | (5, 'poor') | (5, 'poor') | (5, 'poor') | (5, 'poor') |
| (6, 'good') | (6, 'good') | (6, 'good') | (6, 'good') | (6, 'good') | (6, 'good') | (6, 'good') |
| (7, 'good') | (7, 'good') | (7, 'good') | (7, 'good') | (7, 'good') | (7, 'good') | (7, 'good') |
| (8, 'good') | (8, 'good') | **(8, 'poor')** | **(8, 'poor')** | (8, 'good') | (8, 'good') | (8, 'good') |
| (9, 'good') | (9, 'good') | **(9, 'poor')** | **(9, 'poor')** | (9, 'good') | (9, 'good') | (9, 'good') |
| (10, 'good') | (10, 'good') | **(10, 'poor')** | **(10, 'poor')** | **(10, 'poor')** | **(10, 'poor')** | **(10, 'poor')** |
| (11, 'good') | (11, 'good') | **(11, 'poor')** | **(11, 'poor')** | (11, 'good') | **(11, 'poor')** | **(11, 'poor')** |
| (12, 'good') | (12, 'good') | (12, 'good') | (12, 'good') | (12, 'good') | (12, 'good') | (12, 'good') |
| (13, 'good') | **(13, 'poor')** | (13, 'good') | (13, 'good') | (13, 'good') | (13, 'good') | (13, 'good') |
| (14, 'good') | **(14, 'poor')** | (14, 'good') | (14, 'good') | (14, 'good') | (14, 'good') | (14, 'good') |
| (15, 'good') | (15, 'good') | (15, 'good') | (15, 'good') | (15, 'good') | (15, 'good') | (15, 'good') |
| (16, 'poor') | (16, 'poor') | (16, 'good') | (16, 'good') | (16, 'good') | (16, 'good') | (16, 'good') |
| (17, 'poor') | (17, 'poor') | **(17, 'poor')** | **(17, 'poor')** | (17, 'good') | (17, 'good') | (17, 'good') |
| (18, 'poor') | (18, 'poor') | **(18, 'poor')** | **(18, 'poor')** | **(18, 'good')** | **(18, 'good')** | **(18, 'good')** |
| (19, 'poor') | (19, 'poor') | **(19, 'poor')** | **(19, 'poor')** | **(19, 'good')** | **(19, 'good')** | **(19, 'good')** |
| (20, 'poor') | (20, 'poor') | (20, 'good') | **(20, 'poor')** | (20, 'good') | (20, 'good') | (20, 'good') |
| (21, 'poor') | (21, 'poor') | (21, 'good') | **(21, 'poor')** | (21, 'good') | (21, 'good') | (21, 'good') |
| (22, 'poor') | (22, 'poor') | (22, 'poor') | (22, 'poor') | (22, 'poor') | (22, 'poor') | (22, 'poor') |
| (23, 'poor') | (23, 'poor') | (23, 'good') | **(23, 'poor')** | (23, 'good') | (23, 'good') | (23, 'good') |
| (24, 'poor') | (24, 'poor') | **(24, 'poor')** | **(24, 'poor')** | (24, 'good') | (24, 'good') | (24, 'good') |
|  |  | (25, 'good') | (25, 'good') | (25, 'good') | (25, 'good') | (25, 'good') |
|  |  | (26, 'good') | (26, 'good') | (26, 'good') | (26, 'good') | (26, 'good') |
|  |  | (27, 'good') | (27, 'good') | (27, 'good') | (27, 'good') | (27, 'good') |
|  |  | (28, 'good') | **(28, 'poor')** | (28, 'good') | (28, 'good') | (28, 'good') |
|  |  | **(29, 'poor')** | **(29, 'poor')** | **(29, 'poor')** | **(29, 'poor')** | **(29, 'poor')** |
| Wrong number 0 | 3 | 9 | 13 | 8 | 8 | 7 |

**Table S1:** Predicted outcome with larger or smaller patches in term of the field of view. When the results are highlighted in yellow, it means that the new approach made a prediction that is not consistent with the base line procedure. If the results are labeled in red bold font, it means that the prediction is wrong.

| Validation_all_0.25 | Validation_all_0.35 | predict_all_0.25 | predict_all_0.35 | **triple_0.3** |
| --- | --- | --- | --- | --- |
| **(1, 'poor')** | **(1, 'poor')** | (1, 'poor') | (1, 'poor') | **(1, 'good')** |
| (2, 'good') | (2, 'good') | (2, 'poor') | (2, 'poor') | (2, 'poor') |
| (3, 'good') | (3, 'good') | (3, 'poor') | (3, 'poor') | (3, 'poor') |
| (4, 'good') | (4, 'good') | (4, 'poor') | (4, 'poor') | (4, 'poor') |
| (5, 'good') | (5, 'good') | (5, 'poor') | (5, 'poor') | (5, 'poor') |
| (6, 'good') | (6, 'good') | (6, 'good') | (6, 'good') | (6, 'good') |
| (7, 'good') | (7, 'good') | (7, 'good') | (7, 'good') | (7, 'good') |
| (8, 'good') | (8, 'good') | (8, 'good') | (8, 'good') | (8, 'good') |
| (9, 'good') | (9, 'good') | **(9, 'poor')** | **(9, 'poor')** | **(9, 'poor')** |
| (10, 'good') | (10, 'good') | **(10, 'poor')** | **(10, 'poor')** | **(10, 'poor')** |
| (11, 'good') | (11, 'good') | **(11, 'poor')** | **(11, 'poor')** | **(11, 'poor')** |
| (12, 'good') | (12, 'good') | (12, 'good') | (12, 'good') | (12, 'good') |
| **(13, 'poor')** | **(13, 'poor')** | (13, 'good') | (13, 'poor') | (13, 'good') |
| (14, 'good') | (14, 'good') | (14, 'good') | (14, 'good') | (14, 'good') |
| (15, 'good') | (15, 'good') | (15, 'good') | (15, 'good') | (15, 'good') |
| (16, 'poor') | (16, 'poor') | (16, 'good') | (16, 'good') | (16, 'good') |
| (17, 'poor') | (17, 'poor') | (17, 'good') | (17, 'poor') | (17, 'good') |
| (18, 'poor') | (18, 'poor') | **(18, 'poor')** | **(18, 'poor')** | **(18, 'poor')** |
| (19, 'poor') | (19, 'poor') | **(19, 'poor')** | **(19, 'poor')** | **(19, 'poor')** |
| (20, 'poor') | (20, 'poor') | (20, 'good') | (20, 'good') | (20, 'good') |
| (21, 'poor') | (21, 'poor') | (21, 'good') | (21, 'good') | (21, 'good') |
| (22, 'poor') | (22, 'poor') | (22, 'poor') | (22, 'poor') | (22, 'poor') |
| (23, 'poor') | (23, 'poor') | (23, 'good') | (23, 'good') | (23, 'good') |
| (24, 'poor') | (24, 'poor') | (24, 'good') | **(24, 'poor')** | (24, 'good') |
|  |  | (25, 'good') | (25, 'good') | (25, 'good') |
|  |  | (26, 'good') | (26, 'good') | (26, 'good') |
|  |  | (27, 'good') | (27, 'good') | (27, 'good') |
|  |  | (28, 'good') | (28, 'good') | (28, 'good') |
|  |  | **(29, 'poor')** | **(29, 'poor')** | **(29, 'poor')** |

**Table S2:** Keeping patches without T cells in the CH and MG cohort simply lowers down the threshold Rc. When the results are highlighted in yellow, it means that the new approach made a prediction that is not consistent with the base line procedure. If the results are labeled in red bold font, it means that the prediction is wrong.

**3. A modification for easier application.**

We think the process of counting images would bring some barrier to the application of the method because it needs some coding. So we introduce a third class that contains images (15k) that are thrown away in our previous experiment. We randomly select about 30% images as the third class for training and validation. Now, the prediction could be good, poor, or third class. We still use good/(good+poor) to compare with the threshold. With this modification, only the training process needs to count pixels. For the people who would like to use the trained model, the only thing needs to do is cutting images into 64*64 and feed into the network, which could be done by a graphic operation. Details are presented in Table S2 (With “triple” in column name).

**4. Balanced number of patches from patients with the poor/good outcome in the training set.**

In the original training set, there are more patches from patients with good outcome than from patients with poor outcome. Therefore, in the baseline procedure shown in the main text, we chose to quadruplicate patches obtained from patients with poor outcome. This was done to create a balanced dataset. To test whether this operation could introduce bias in the final prediction or over-fitting of the data, we randomly remove some good-outcome patients from the training set and move some patients with the poor outcome to the training set. The details of these sets are presented in Table S3. Now with a balanced training-set, the predictive power of this approach is (slightly) worse than our original one - see Table S4 for details. Since there was no substantial difference between the two balancing methods, overfitting was less of a concern. Therefore, we prefer our original approach, which make use of images from one cohort and then has the trained neuron network tested with an independent cohort.

|  | Patient ID in the train set | Patient ID in the test set |
| --- | --- | --- |
| CH cohort  Patients with the good outcome | 3,4,7,10,11 | 1,2,5,6,8,9,12,13,14,15 |
| CH cohort  Patients with the poor outcome | 16,17,18,19,20,21,22,23,24 | None |
| MG cohort  Patients with the good outcome | None | 6-21,23-29 |
| MG cohort  Patients with the poor outcome | 1,2,3,4 | 5,22 |

**Table S3:** Patients selected for balanced number of patches in the training-set and patients left for the test set.

| CH cohort | | | MG cohort | | | | | |
| --- | --- | --- | --- | --- | --- | --- | --- | --- |
| Patient ID | Prediction | Main-text approach | Patient ID | Prediction | Main-text approach | Patient ID | Prediction | Main-text approach |
| 1 | **poor** | good | 5 | poor | poor | 17 | good | good |
| 2 | good | good | 6 | good | good | 18 | **poor** | **poor** |
| 5 | good | good | 7 | good | good | 19 | **poor** | **poor** |
| 6 | good | good | 8 | **poor** | good | 20 | good | good |
| 8 | good | good | 9 | **poor** | **poor** | 22 | poor | poor |
| 9 | good | good | 10 | **poor** | **poor** | 23 | good | good |
| 12 | good | good | 11 | **poor** | **poor** | 24 | **poor** | good |
| 13 | good | good | 12 | good | good | 25 | good | good |
| 14 | good | good | 13 | good | good | 26 | good | good |
| 15 | good | good | 14 | good | good | 28 | good | good |
|  |  |  | 15 | good | good | 29 | **poor** | **poor** |
|  |  |  | 16 | good | good |  |  |  |

**Table S4**: Predictions with balanced number of patches in the training-set and comparison with original results. When the results are highlighted in yellow, it means that the new approach made a prediction that is not consistent with the base line procedure. If the results are labeled in red bold font, it means that the prediction is wrong.

**5. The hierarchical clustering and principal component analysis of the CH cohort using various clinical characteristics.**

As shown in Table S5, we collected as much information as we could for the CH cohort. Next, we converted the textual information into numerical values for input into the hierarchical clustering analysis or principal component analysis (Table S6). As demonstrated in Figure S2A, we did not observe obvious clustering of patients with good/poor outcome via hierarchical clustering analysis. For the PCA, as shown in Figure S2B, with a proper scaling of the variables, in the PC1 and PC2 plane we could achieve a separation between the poor- and good- outcome patient groups with an accuracy of 83%. The most important factors are CD8_in/CD8_total and CD8_in/PanCK, which is consistent with the results shown in main Figure 3A. In addition, the size of tumor as well as the patient ethnicity are the other two major factors that can help to separate the two groups. Nevertheless, these analyses cannot achieve the perfect separation of the two groups for the CH cohort, which was realized by our deep-learning approach. Also, if we do not scale the variables properly, the separation between the two group becomes worse (Figure S2B).

| Patient ID | TNM | Grade | Age at Sx | Stage | Ethnicity | Size (cm) | Chemo | Radiation | CD8_in/  CD8_total | CD8_in/PanCK |
| --- | --- | --- | --- | --- | --- | --- | --- | --- | --- | --- |
| 1 | T1cN0(i-)(sn)MX | 3 | 72 | IA | White/  Caucasian | 1.3 | Declined | xRT | 0.0444 | 0.0054 |
| 2 | T2N0MX | 3 | 44 | IIA | White/  Caucasian | 3 | AC | xRT | 0.6882 | 0.0784 |
| 3 | T3N0MX | 3 | 57 | IIB | Asian | 3.2 | AC | xRT | 0.0174 | 0.0092 |
| 4 | T2N0MX | 3 | 58 | IIA | White/  Caucasian | 4.2 | ACT | Unknown | 0.3126 | 0.0316 |
| 5 | T2N2aMX | 3 | 53 | IIIA | White/  Caucasian | 4.5 | ACT | xRT | 0.5666 | 0.0522 |
| 6 | T2N0MX | 3 | 47 | IIA | Asian | 4.7 | ACT | xRT | 0.4345 | 0.0486 |
| 7 | T1cN1M0 | 2 | 60 | IIB | White/  Caucasian | 1.8 | ACT | None | 0.2213 | 0.0292 |
| 8 | T2N0MX | 3 | 54 | IIA | White/  Caucasian | 3.2 | ACT | xRT | 0.1094 | 0.0122 |
| 9 | T2N0M0 | 3 | 27 | IIA | White/  Caucasian | 2.2 | ACT | Unknown | 0.1675 | 0.0232 |
| 10 | T2N0M0 | 3 | 64 | IIA | White/  Caucasian | 2.5 | ACT | xRT | 0.1832 | 0.0038 |
| 11 | T2N1aMX | 3 | 50 | IIB | White/  Caucasian | 4 | ACT | xRT | 0.0750 | 0.0122 |
| 12 | T2N0(i-)(sn)MX | 3 | 43 | IIA | Other | 3.5 | ACT | Unknown | 0.5515 | 0.0535 |
| 13 | T2N0MX | 3 | 67 | IIA | White/  Caucasian | 2.5 | Unknown | Unknown | 0.0354 | 0.0039 |
| 14 | T2N0MX | 2 | 57 | IIA | Asian | 3.2 | ACT | None | 0.0083 | 0.0023 |
| 15 | T2N2aMX | 3 | 54 | IIIA | White/Caucasian | 2.8 | ACT | xRT | 0.0471 | 0.0027 |
|  |  |  |  |  |  |  |  |  |  |  |
| 16 | T1cN0MX | 3 | 56 | IA | African Amer | 1.6 | ACT | xRT | 0.0073 | 0.0002 |
| 17 | T1cN0(i-)MX | 3 | 57 | IA | Hispanic or Latino | 1.5 | ACT | xRT | 0.0416 | 0.0017 |
| 18 | T2 N0(i-) MX | 3 | 79 | IIA | Asian | 3.3 | Declined | xRT | 0.0894 | 0.0084 |
| 19 | T2N1aMX | 3 | 46 | IIB | Other | 3.2 | TC | None | 0.0516 | 0.0022 |
| 20 | T3N2aMX | 3 | 35 | IIIA | African Amer | 1.2 | AC | xRT | 0.1117 | 0.0004 |
| 21 | T2N0(i-)(sn)MX | 3 | 71 | IIA | White/  Caucasian | 2.5 | ACT | Unknown | 0.0091 | 0.0003 |
| 22 | T2pN0MX | 3 | 46 | IIA | Asian | 2.1 | ACT | xRT | 0.0197 | 0.0012 |
| 23 | T1cN0(i-)MX | 3 | 65 | IA | White/  Caucasian | 1.1 | Tamoxifen* | xRT | 0.0780 | 0.0019 |
| 24 | T2N0(i-)M1 | 3 | 46 | IV | White/  Caucasian | 3.8 | Unknown | Unknown | 0.0414 | 0.0021 |

**Table S5:** Clinical characteristics of patients from the CH cohort. *For patient 23, she had a history of bilateral breast cancer: the subtype of tumor from the left breast is TNBC and that from the right is ER^+^HER2^-^. Adjuvant hormonal therapy was given for the right breast ER+ disease. And we used the specimen from the left breast which is TNBC.

| Patient ID | T | N | M | Gra-de | Age at Sx /10 | Stage | Ethnicity | Size (cm) | Chemo | Radiation | CD8_in/  CD8_total x 10 | CD8_in/PanCK x 100 |
| --- | --- | --- | --- | --- | --- | --- | --- | --- | --- | --- | --- | --- |
| 1 | 1 | 0 | 2 | 3 | 7.2 | 1 | 4 | 1.3 | 0 | 1 | 0.444 | 0.54 |
| 2 | 2 | 0 | 2 | 3 | 4.4 | 3 | 4 | 3 | 3 | 1 | 6.882 | 7.84 |
| 3 | 3 | 0 | 2 | 3 | 5.7 | 4 | 2 | 3.2 | 3 | 1 | 0.174 | 0.92 |
| 4 | 2 | 0 | 2 | 3 | 5.8 | 2 | 4 | 4.2 | 1 | 2 | 3.126 | 3.16 |
| 5 | 2 | 2 | 2 | 3 | 5.3 | 5 | 4 | 4.5 | 1 | 1 | 5.666 | 5.22 |
| 6 | 2 | 0 | 2 | 3 | 4.7 | 3 | 2 | 4.7 | 1 | 1 | 4.345 | 4.86 |
| 7 | 1 | 1 | 0 | 2 | 6 | 4 | 4 | 1.8 | 1 | 0 | 2.213 | 2.92 |
| 8 | 2 | 0 | 2 | 3 | 5.4 | 3 | 4 | 3.2 | 1 | 1 | 1.094 | 1.22 |
| 9 | 2 | 0 | 0 | 3 | 2.7 | 3 | 4 | 2.2 | 1 | 2 | 1.675 | 2.32 |
| 10 | 2 | 0 | 0 | 3 | 6.4 | 3 | 4 | 2.5 | 1 | 1 | 1.832 | 0.38 |
| 11 | 2 | 1 | 2 | 3 | 5 | 4 | 4 | 4 | 1 | 1 | 0.75 | 1.22 |
| 12 | 2 | 0 | 2 | 3 | 4.3 | 3 | 3 | 3.5 | 1 | 2 | 5.515 | 5.35 |
| 13 | 2 | 0 | 2 | 3 | 6.7 | 3 | 4 | 2.5 | 2 | 2 | 0.354 | 0.39 |
| 14 | 2 | 0 | 2 | 2 | 5.7 | 3 | 2 | 3.2 | 1 | 0 | 0.083 | 0.23 |
| 15 | 2 | 2 | 2 | 3 | 5.4 | 5 | 4 | 2.8 | 1 | 1 | 0.471 | 0.27 |
|  |  |  |  |  |  |  |  |  |  |  |  |  |
| 16 | 1 | 0 | 2 | 3 | 5.6 | 1 | 0 | 1.6 | 1 | 1 | 0.073 | 0.02 |
| 17 | 1 | 0 | 2 | 3 | 5.7 | 1 | 1 | 1.5 | 1 | 1 | 0.416 | 0.17 |
| 18 | 2 | 0 | 2 | 3 | 7.9 | 3 | 2 | 3.3 | 0 | 1 | 0.894 | 0.84 |
| 19 | 2 | 1 | 2 | 3 | 4.6 | 4 | 3 | 3.2 | 1 | 0 | 0.516 | 0.22 |
| 20 | 3 | 2 | 2 | 3 | 3.5 | 5 | 0 | 1.2 | 3 | 1 | 1.117 | 0.04 |
| 21 | 2 | 0 | 2 | 3 | 7.1 | 3 | 4 | 2.5 | 1 | 2 | 0.091 | 0.03 |
| 22 | 2 | 0 | 2 | 3 | 4.6 | 3 | 2 | 2.1 | 1 | 1 | 0.197 | 0.12 |
| 23 | 1 | 0 | 2 | 3 | 6.5 | 1 | 4 | 1.1 | 1 | 1 | 0.78 | 0.19 |
| 24 | 2 | 0 | 1 | 3 | 4.6 | 7 | 4 | 3.8 | 2 | 0 | 0.414 | 0.21 |

**Table S6:** To perform statistical analysis, the corresponding information is converted from texts to numbers.


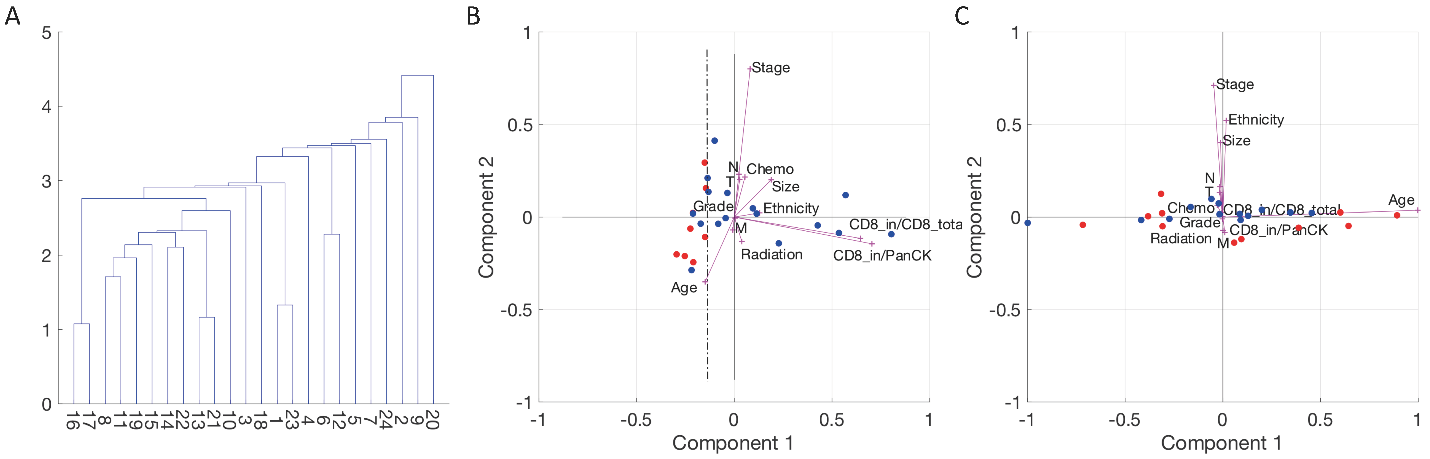


**Figure S2:** Hierarchical clustering (A) and principal component analysis (B and C) of the clinical characteristics of patients in the CH cohort. In B and C, the principle components are labelled as x and y axis. The mapping of characteristic vectors on the PC are labeled in cyan. Blue (red) dots represent patients with the good (poor) outcome. The dash-dotted line in B marks the best separation threshold between the two group with the help of PC1. The difference between B and C is that age, CD8_in/CD8_total, CD8_in/PanCK were not scaled as shown in Table S5.

**6. Additional information regarding the computation.**

For the results demonstrated in main text, the deep-learning codes run under the platform with python 3.5, mxnet 1.1.0, cuda 7.5. And the results demonstrated in SI run on the platform with python 3.6., mxnet 1.10, cuda 9.0. We should point out that classification for three patients in the MG cohort (P1, P2 and P24) can be marginal, which can be affected by randomness embedded in the choice of training set and computational platform. It is therefore important to analyze the training data and derive associated thresholds using the same computational platform as will then be applied to test data.
